# Enhancing wet phosphoric acid production efficiency with vanadium catalyst waste derived silica

**DOI:** 10.1016/j.heliyon.2024.e38968

**Published:** 2024-10-05

**Authors:** Islaam Anouar, Rim Jouraiphy, Hamid Mazouz, Nils Haneklaus, Samia Yousfi, Mouna L. Bouamrani

**Affiliations:** a Moroccan Foundation for Advanced Science, Innovation & Research, Rabat, Morocco; b Mohammed VI Polytechnic University (UM6P), Lot 660 Hay Moulay Rachid, Ben Guerir, 43150, Morocco; c Laboratory of Analytical Chemistry and PhysicoChemistry of Materials, Department of Chemistry, Faculty of Sciences Ben M'Sik, University Hassan II, Casablanca, Morocco; d Engineering, Industrial Management, and Innovation Laboratory, Faculty of Science and Techniques, University HASSAN I, Settat, Morocco; e Center of Expertise for Phosphate (CEPH), Mohammed VI Polytechnic University (UM6P), Ben Guerir, 43150, Morocco; f Td-Lab Sustainable Mineral Resources, Universität für Weiterbildung Krems, Dr.-Karl-Dorrek-Straße 30, 3500, Krems an der Donau, Austria; g North-West University, Unit for Energy and Technology Systems - Nuclear Engineering, 11 Hoffman Street, Potchefstroom, 2520, South Africa

**Keywords:** Phosphogypsum, Crystallization, Active silica, Vanadium catalyst, Wet phosphoric acid (WPA)

## Abstract

Filtration of phosphogypsum (PG) is an important step in the production of wet phosphoric acid (WPA). In recent years, the phosphate rocks used for WPA production in Morocco started showing deficiencies in terms of their reactive silica content that affects PG crystallization and as such decreases PG filtration efficiency and subsequently the overall efficiency of the WPA production. In this work reactive silica waste from spent vanadium catalysts, used in the production of sulfuric acid for WPA production, was added to the digestion process in an attempt to increase WPA production efficiencies. The laboratory work presented here shows that the added silica improved the quality of the produced dihydrate WPA by 6 %. Besides, the chemical yield of the reaction was improved by 5 % as a result of the shape-change of the PG crystals that allowed for better filtration. The results of this work are promising and we strongly recommend testing this approach on larger pilot- and ultimately industrial scale.

## 1 Introduction

**Fig. 1:**
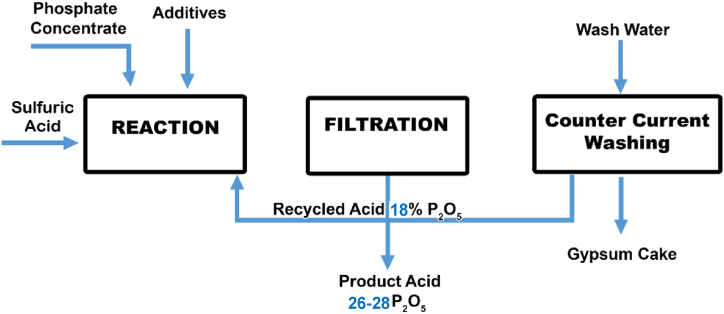
Simplified overview of the dihydrate process for the production of wet phosphoric acid (WPA) with sulfuric acid.

Phosphate rock is among the five most mined ores on earth. The material is largely used for mineral fertilizer production and is thus closely connected to global food security. Thermal processes and wet phosphoric acid (WPA) processes can be used to process phosphate rock to phosphoric acid and ultimately mineral fertilizers [[1], [2], [3]]. Globally more than 90 % of all phosphate rock is processed using the WPA process with sulfuric acid that is schematically shown in Fig. 1 [4,5].

The dehydrate process usually leads to an acid with a $P_{2}O_{5}$ content ranging from 26 to 28 % [6,7]. The most critical step in WPA production is the separation of the solid phosphogypsum (PG) byproduct from the liquid WPA product by filtration [3,8]. This step is largely affected by the attack conditions, crystallization of the PG, and the quality of the used phosphate rock concentrate [[9], [10], [11]].

If phosphate rock contains high concentrations of silica (with a SiO₂/F ratio exceeding 0.53), it leads to the nearly complete complexation of fluorine into H₂SiF₆. To mitigate the corrosive effects of fluorine, it is recommended to maintain a minimum silica content of 3 %. However, while excess silica does not adversely affect the reaction, it can cause abrasion to the equipment. In industrial production the SiO₂/F ratio (or reactive F/SiO₂ content) is regulated by adding reactive silica to the phosphate concentrate to ensure that the ratio stays slightly above 0.53 so that calcium sulfate crystals with desirable dimensions for filtration are produced [[12], [13], [14]].

Silica is commonly found as quartz or within the clay present in phosphate rock, either as active silica or soluble silica. During the reaction process, fluorine reacts with the silica to form hexafluorosilicic acid (H₂SiF₆), which (i) enhances crystallization, (ii) improves chemical yield by minimizing losses of water-soluble and syncrystallized P₂O₅, and (iii) reduces the solid content and CaO levels in the resulting acid [11,15,16].

Many works have tested different crystal modifiers for crystallization of calcium sulfate dihydrate, such as organic additives and gelatin [4,17,18], aluminum sulfate, clay (calcined or untreated) [10], active silica, active charcoal, aluminum hydroxide, manganese dioxide, pearlite [10,19], polymers [[20], [21], [22]], surfactants [10,23,24], phosphonates [24,25], foreign ions [26,[27], [28]], and carboxylic acids [25,29]. In addition, different works have investigated the crystallization of other calcium salts such as calcium oxalate and calcium phosphate with and without additives at different levels of supersaturation [[30], [31], [32], [33]].

Silica has proven to be a particularly useful additive in WPA production, and the material could even be provided inexpensively from materials that are presently considered a waste. Arhouni et al. [34] and Hakkar et al. [35] did for instance successfully test the use of fly ash as an inexpensive source of silica to decrease the radioactivity of PG and increase the share of rare earth elements, commonly found in phosphate rock, transferring from the phosphate rock raw material to the liquid WPA from where they could be recovered more easily than from the solid PG [36,37].

For the production of the sulfuric acid used in the WPA production and here specifically the conversion of sulfur dioxide to sulfur trioxide, vanadium catalysts are used that are largely composed of silica. The main elemental composition of such catalysts was for instance reported by Erust et al. [38] to contain 61.04 % SiO_2_, 5.71 % V_2_O_5_, 1.89 % Al_2_O_3_ and 1.17 % Fe_2_O_3_. Vanadium recovery from such catalysts is an active field of research and possible approaches have for instance been reported by Li et el [39]. and Romanovskaia et al. [40]. The utilization of the silica from those catalysts has to the best of our knowledge not been considered yet and its potential use as an inexpensive additive to improve WPA production is for the first time described in this work.

## 2 Materials and methods

### 2.1 Materials

The phosphate slurry used in this study was obtained from the Khouribga region in Morocco. As a result of the large amounts of phosphate rock mined here the region is one of the most relevant phosphate extraction sites in the world. The slurry had a solid content of 60 %. Industrial-grade chemical reagents, such as 65 % concentration sulfuric acid (H₂SO₄), 18 % concentration phosphoric acid (H₃PO₄) and tab water were used in the experiments to replicate industrial production conditions as closely as possible. The reactive silica was sourced from old vanadium catalysts used for sulfuric acid production in the fertilizer industry in Morocco.

### 2.2 Methods

**Fig. 2:**
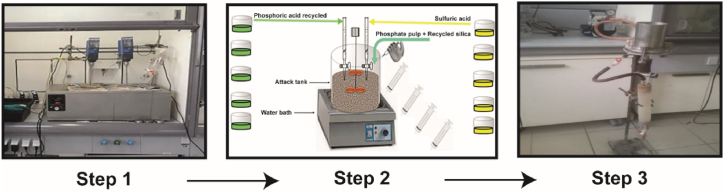
Schematic representation of the experimental setup for the production of wet phosphoric acid (WPA) used in this work.

The experimental approach in this study was centered around two main stages and is illustrated in Fig. 2. Initially, there was a focus on rectifying the reactive silica level, followed by the sequential implementation of the WPA production process under well-defined attack and filtration conditions. The processing at laboratory scale is carried out according to the dihydrate process, using a 2 L metallic reactor equipped with mechanical stirring and immersed in a water bath so that the temperature can be maintained at 80 °C. Although the work was done on laboratory scale, the same reagents also used on industrial scale in Morocco, namely 65 % sulfuric acid, phosphate pulp with a 60 % solids content, and recycled phosphoric acid with a 18 % P_2_O_5_ content were used to replicate industrial conditions as closely as possible. Specifically, the operational conditions were as following: 80 °C reaction temperature, 30–35 % slurry solids content, 25–30 g/L free sulfate concentration, and 1.240–1.260 kg/L recycled phosphoric acid density. The reagents were gradually injected into the phosphate pulp during constant stirring. The reaction time was 2 h and 15 min. The additives, dissolved beforehand, were added during the reinjection. The reaction mixture, known as slurry, was then left for 1 h under constant agitation to allow maturation. After maturation, the slurry was filtered using a Buchner vacuum filter. Reactions of the manufacturing process are provided in Equations (1), (2), (3), (4), (5), (6), (7), (8), (9), (10)).

It is noteworthy that the reactive silica content of the phosphate used here is insufficient for a good reaction performance. It is therefore necessary to add silica until an appropriate ${\text{SiO}}_{2}$/F ratio is reached in Equation (1).

(1) SiO_2(s)_+6HF_(aq)_→H_2_[SiF_6_]_(aq)_+2H_2_O

(2) $$n\left({\text{SiO}}_{2}\right)=\frac{1}{6}\;n\left(\text{HF}\right)↔\frac{m\left({\text{SiO}}_{2}\right)}{M\left({\text{SiO}}_{2}\right)}=\frac{m\left(\text{HF}\right)}{6M5\text{HF})}$$

(3) $$\frac{m\left({\text{SiO}}_{2}\right)}{m\left(\text{HF}\right)}=\frac{M\left({\text{SiO}}_{2}\right)}{6M\left(\text{HF}\right)}=\frac{60}{120}=0.5$$

(4) $$m\left({\text{SiO}}_{2}\right)=0.5\times m\left(\text{HF}\right)$$

(5) $$H^{+}+F^{-}\to HF$$

(6) $$n\left(H^{+}\right)=n\left(F^{-}\right)↔m\left(\text{HF}\right)=\frac{m\left(F^{-}\right)\times M\left(\text{HF}\right)}{M\left(F^{-}\right)}$$

(7) $$m\left({\text{SiO}}_{2}\right)=0.5\times \frac{m\left(F^{-}\right)\times M\left(\text{HF}\right)}{M\left(F^{-}\right)}$$

that can be expressed as:

(8) $$m\left({\text{SiO}}_{2}\right)=m{\left({SiO}_{2}\right)}_{\text{real}\;}+m{\left({SiO}_{2}\right)}_{\;\text{to}\;\text{add}}$$

(9) $${m\left({\text{SiO}}_{2}\right)}_{\text{to}\;\text{add}}=m\left({\text{SiO}}_{2}\right)\;–\;m{\left({SiO}_{2}\right)}_{\text{real}}$$

(10) $${m\left({\text{SiO}}_{2}\right)}_{\text{to}\;\text{add}}=0.5\times \frac{m\left(F-\right)\times M\left(\text{HF}\right)}{M\left(F-\right)}-m{\left({SiO}_{2}\right)}_{\text{real}}$$

with M as the Molar mass, m (${\text{SiO}}_{2}$) as the mass of silica, according to equation (1). m (${\text{SiO}}_{2}$) also represents the difference between the existing silica (${\text{SiO}}_{2R}$) in the phosphate and the silica that must be added (${\text{SiO}}_{2}$_to add_), in order to correct the real (or present) silica content so that the desired ratio required for a suitable reaction is reached. The silica that needs to be added is denoted here as m (${\text{SiO}}_{2}$) _to add_ while m (${\text{SiO}}_{2}$) _real_ represents the silica content present in the phosphate slurry.

### 2.3 Characterizations

The particle size distribution of the phosphate rock concentrate was determined using a Malvern Mastersizer 2000 particle size analyzer. The elemental composition of the crude phosphate and the recycled silica additive was determined through inductively coupled plasma atomic emission spectroscopy (ICP-AES) with a Thermo Jarrell-Ash IRIS. Scanning electron microscopy (SEM) and energy dispersive X-ray spectroscopy (EDX) were performed with a FEI Quanta 450 FEG. The morphology and texture of the PG were assessed using optical microscopy with a Leica DM 2500. N2 adsorption-desorption analysis was performed using a micromeritics surface area analyzer at 77 K, employing the Brunauer-Emmett-Teller (BET) and Barrett-Joyner-Halenda (BJH) methods to calculate the specific surface area, pore volume, and pore size distributions. Rheological measurements, including viscosity and shear stress, of the phosphate pulp (with and without doping) were carried out using an automatic rotational rheometer (Anton-Paar). In the used setup, the rotor was attached to the spindle, and the samples were poured into the cup. The used rheometer was equipped with a thermostat for adjusting the sample temperature during the rheological measurements. The rotation was ranged from high to low shear rates to capture various flow conditions. The density of the WPA was determined using a Densitopro (Mettler Toledo) densimeter. The P_2_O_5_ concentration was determined using a complexation method based on UV spectroscopy. The viscosity of the phosphoric acid was determined using a rotary PCE-RVI 2 viscometer.

## 3 Results and discussion

### 3.1 Raw materials characterization

**Fig. 3:**
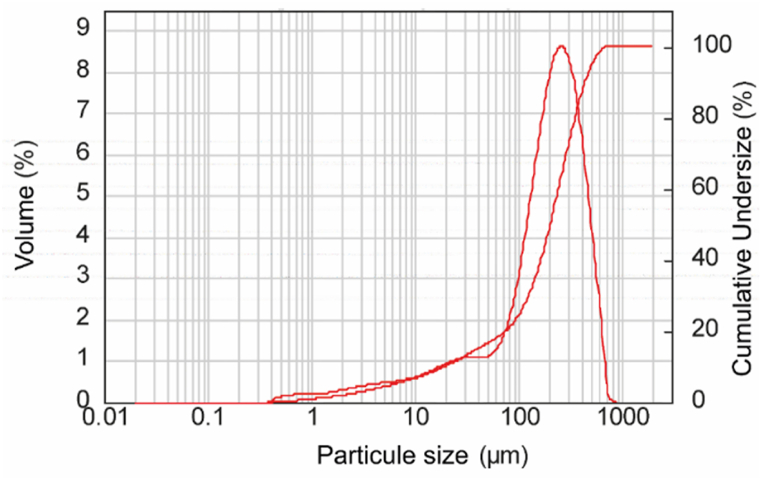
The particles size distribution of Khouribga phosphate ore.

The results of the particle size distribution of the phosphate rock are shown in Fig. 3. 14.9 % of the measured particles were below 40 μm and 95.3 % of all measured particles were between 80 and 800 μm.

The elemental composition of the received Khouribga phosphate ore is presented in Table 1. The dry phosphate rock used in this study shows a relatively high P_2_O_5_ content (30.5 %) and relatively low levels of ${\text{Al}}_{2}O_{3}$ (0.3 %). The total silica content was determined to be 2.6 % of which 1.1 % can be considered reactive silica.

**Table 1:** Chemical analysis of the Khouribga phosphate ore measured by ICP-OES.

Component	P_2_O_5_	CaO	SiO_2T_	SiO_2R_	Al_2_O_3_	Fe_2_O_3_	F^−^	MgO	SO_3_	CO_2_
Concentration (%)	30.5	50.4	2.6	1.1	0.3	0.2	3.4	0.5	2.1	7.4

**Fig. 4:**
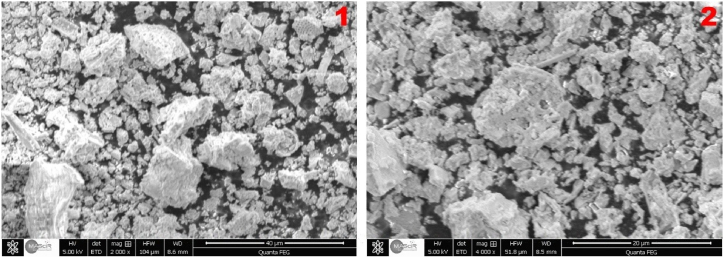
SEM photomicrographs of new silica resource with 2000 (1) and 4000 (2) magnification (40 μm and 20 μm).

SEM-EDX analysis was used to determine the shape and composition of the reactive silica additive. Fig. 4 shows that the additive has a heterogeneous microstructure consisting of crystallites that showed a length of up to 10 μm.

The EDX analysis indicated the presence of a variety of elements (4.88 % C, 49.99 % O, 49.61 % Si, 1.06 % K, 0.61 % Fe, 0.13 % V) that could also be detected using additional ICP-AES analysis (Table 2).

**Table 2:** Elemental composition of additive measured by ICP-AES.

Component	SiO_2T_	SiO_2R_	Al_2_O_3_	Fe_2_O_3_	F^−^	K_2_O	CaO	V
Concentration (%)	48.2	47.2	1.3	0.5	0.3	0.5	0.3	0.3282

**Fig. 5:**
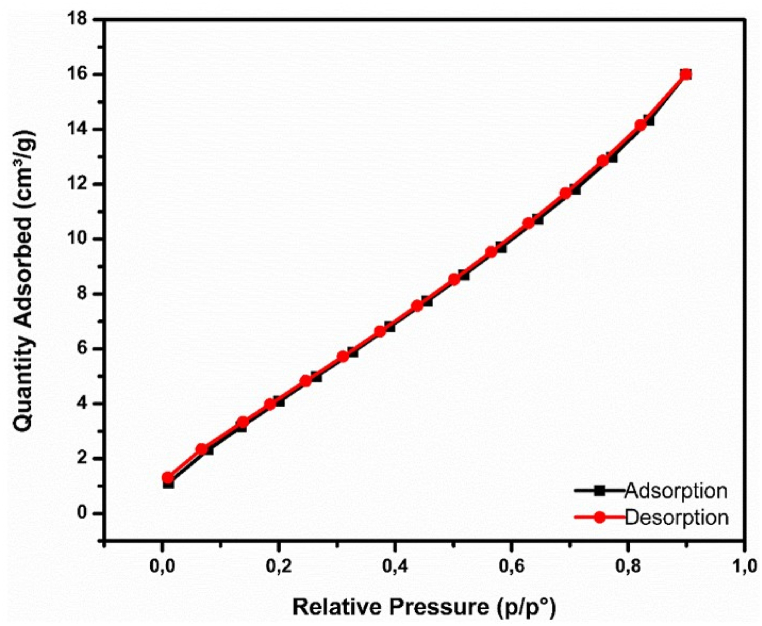
Curve of the quantity of nitrogen adsorbed/desrobed by the silica byproduct.

The porosity, the specific surface area, and the isotherms adsorption/desorption of nitrogen (N_2_) at 77 K by the silica byproduct are shown in Table 3 and Fig. 5.

**Table 3:** Characterization of the porosity and the specific surface area of silica additive.

BET Surface Area	Average Pore Volume	Average Pore Size	Average Nanoparticle Size
20.5454 m^2^/g	0.0248 cm^3^/g	4.8251 nm	737.7348 nm

Pore sizes can loosely be divided into three categories: micropores (size <2 nm), mesopores (size between 2 nm and 50 nm), and macropores, (size >50 nm) [41]. The recycled silica mostly showed mesopores (size between 2 nm and 50 nm), with an average pore volume that did not exceed 0.0248 cm³/g. The measured pore diameters were very close to 2 nm and the results indicated that these relatively small pore sizes did not negatively affect the PG filtration as the porosity was low.

Generally, the various physical and chemical properties of the phosphate pulp have significant influence on its rheology. These parameters include density, solids concentration (or solid content), particle size distribution, and temperature [[42], [43], [44], [45], [46]]. In this study, phosphate pulp behaved like a pseudoplastic fluid characterized by its density and apparent viscosity. During the experiments the slurry flow regime was always kept in turbulence to prevent sedimentation of solid aggregates at the bottom of the reaction container. The same is done during industrial WPA production where sediments could otherwise accumulate and block pipelines or otherwise interfere with the WPA production.

During the preparation of phosphate pulp (with and without doping), it is necessary for the solid phosphate concentration to be within the range recommended by the pipeline designer (usually 50–60 % solid content is desirable). The viscosity should, however, be minimal to facilitate storage and transportation of the suspension via pipelines [[42], [43], [44], [45], [46]].

**Fig. 6:**
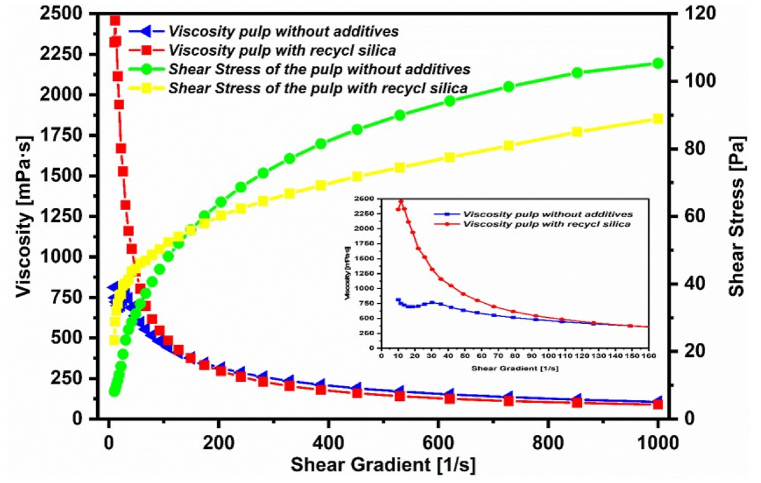
Viscosity and shear stress of the two pulp samples (with and without the addition of recycled silica).

Fig. 6 indicates that the phosphate slurry behaved like a pseudoplastic fluid with a flow threshold. The phosphate pulp doped with the silica byproduct was more viscous than the undoped mixture. It is noteworthy that starting from a shear gradient of 120, the viscosity of both slurries was very similar, indicating no detectable disadvantage (with regards to viscosity) from adding the catalyst waste silica source. The fine particles of the silica were obviously a clear advantage and it was already hypothesized that they would not negatively affect the viscosity while providing relatively large surface areas available for reactions.

### 3.2 Effects of the additives on the process efficiency

The tests reported here were carried out with silica byproduct concentrations of 3.6 kg, 4.0 kg, 5.0 kg and 6.0 kg per metric ton of $P_{2}O_{5}$. Two control experiments were also conducted without the addition of byproduct silica as described by Manar [11]. Experiments without the silica byproduct had the following drawbacks:

• The efficiency of the process was too low with yields less than 90 %;
• Relatively high losses in terms of water soluble material that were greater than 0.4 %;
• It was challenging to obtain WPA with sufficiently high quality;
• Increased levels of free sulfates were detected in the WPA;
• The PG crystal shape was not ideal resulting in lower than desired filterability efficiencies.

The experiments were conducted adding the recycled silica just before the attack stage to determine its effect on the WPA production. The tests without and with additives were carried out at reaction temperatures between 78 °C and 82 °C. The solid content of the pulp was 58–60 % and the density of the return acid was equal to 1160–1170 kg/L. An overview of the test conditions and the test results is presented in Table 4.

**Table 4:** Overview of the test conditions and results.

Test Conditions	Without additives	Without additives	Recycled silica 3.6 kg/T of $P_{2}O_{5}$ equivalent 0.36 %	Recycled silica 4.0 kg/T of $P_{2}O_{5}$ equivalent 0.4 %	Recycled silica 5.0 kg/T of $P_{2}O_{5}$ equivalent 0.5 %	Recycled silica 6.0 kg/T of $P_{2}O_{5}$ equivalent 0.6 %
N° tests	1	2	3	4	5	6
$P_{2}O_{5}$ unattached	0.25	0.22	0.16	0.19	0.2	0.18
$P_{2}O_{5}$ Sy crystallized	0.6	0.58	0.55	0.58	0.54	0.61
$P_{2}O_{5}$ water-soluble	0.41	0.42	0.22	0.26	0.31	0.29
Attack yield (%)	96.6	96.9	97.2	96.8	96.1	96.2
Chemical yield (%)	86.9	90.2	92.8	91.5	82.7	86.5
Filterability efficiency (%)	89.8	93.9	96.2	94.1	86.3	89.9
Filterability ($T\;P_{2}O_{5}$/L)	5.5	5.9	7.8	7.3	6.8	6.6
Acid density (kg/L)	1270	1284	1320	1312	1308	1297
$P_{2}O_{5}$ acid (%)	25.0	26.9	31.6	30.3	27.1	27.0
Free sulfate (g/L)	20.9	24.0	26.4	25.6	16.0	23.0
Viscosity of the WPA (mPa.s)	3.3	3.8	4.2	4.3	4.5	4.6

**Fig. 7:**
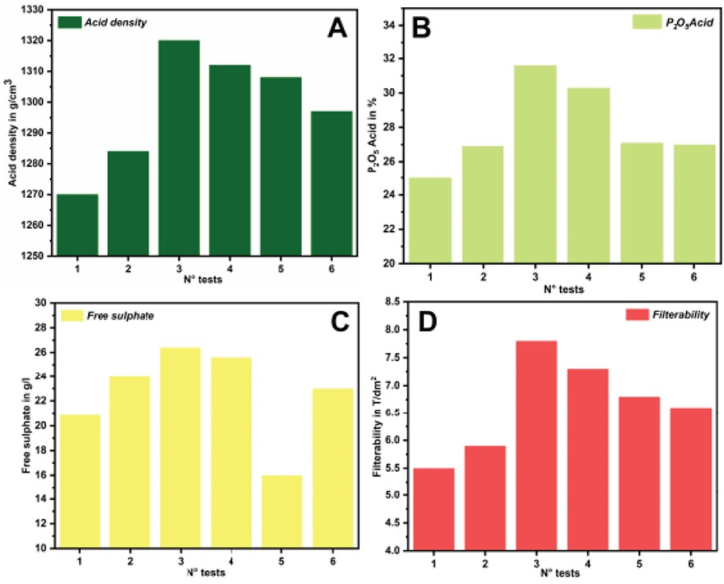
Variation of % $P_{2}O_{5}$ , density, free sulfate and filterability at different quantities of silica byproduct.

Fig. 7A and B show the successful improvement of the density and $P_{2}O_{5}$ concentration of the WPA before and after the addition of silica. The addition of the silica byproduct positively improved of the quality of the WPA in a way that the density could be increased by nearly 50 g/cm³ and the $P_{2}O_{5}$ concentrations was increased by 6 %. These results are in good agreement with those obtained by Manar [11] who considered byproduct silica addition from alumina oxide. Higher WPA densities are usually a good sign since they are linked to higher ${\;P}_{2}O_{5}$ concentrations in the WPA. It is generally important though that the concentration of free sulphates does not exceed a maximum of 25 g/L. Here again the silica byproduct additives are useful as indicated in Fig. 7C. Lastly the filterability of PG was also improved through the addition of the silica byproduct as indicated in Fig. 7D. This work found that the addition of 3.6 kg per ton $P_{2}O_{5}$ seemed to be particularly beneficial resulting in an improvement of about 1.5 t of $P_{2}O_{5}$/m^2^ per day. It is noteworthy that this ratio might be different for other phosphate rocks with varying impurities.

**Fig. 8:**
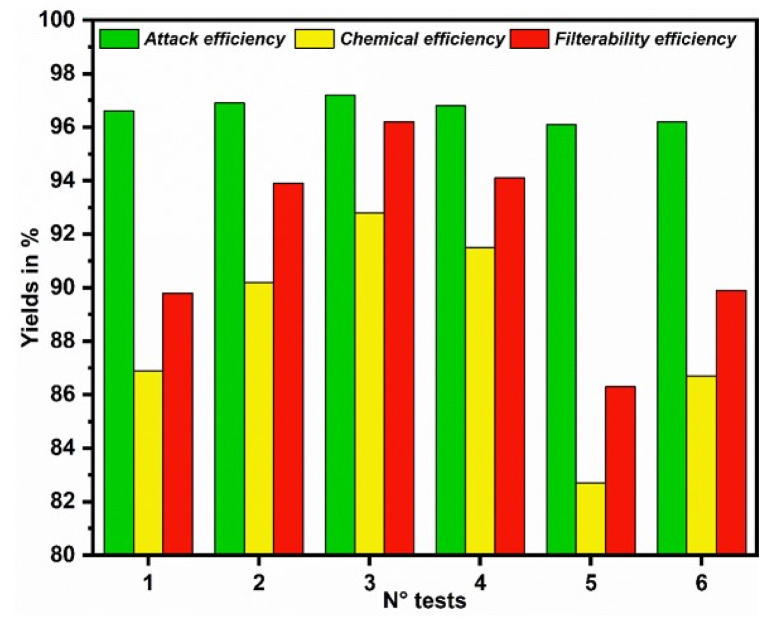
Yield curves of the attack-filtration reactions with different concentrations of the silica byproduct.

According Manar [11] and Omri et al. [47], the addition of silica improves PG filtration. Specifically, the authors reported that the density of the WPA and its $P_{2}O_{5}$ content as well as the yields of the attack reaction (attack efficiency, chemical yield, and filtration efficiency) could be improved. Fig. 8 shows the yields of the attack reactions under different experimental conditions. The results reveal that, the addition of 3.6 kg silica byproduct per ton $P_{2}O_{5}$ significantly increased the attack-, reaction-, and filtration efficiency. With higher concentrations of silica additives, the measured efficiencies went down again, and could even reach levels lower than those of experiments without any silica additives.

**Fig. 9:**
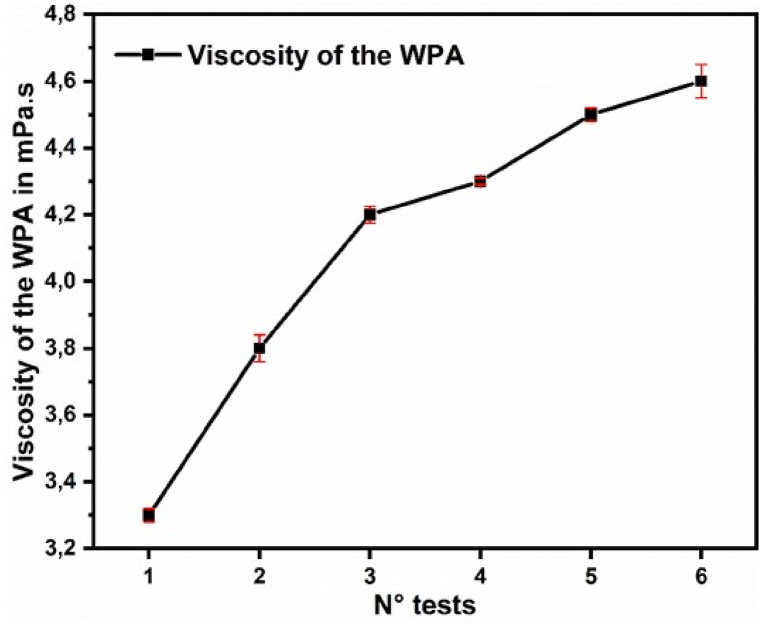
Effect of silica by product addition on the viscosity of the produced.

Fig. 9 shows how the viscosity of the produced WPA increased with increasing amounts of silica byproduct added to the mixture.

### 3.3 Effect of additives on the morphology of the PG crystals

It is well-known that the size and shape of the PG crystals are the most important factors that affect the PG filtration efficiency. For better filtration, it is desirable to have larger crystals of uniform size. During industrial WPA production additives ensure the stability of the reaction, the kinetics of the crystal growth and that the size and the shape of the crystals allow for maximum PG filtration efficiency.

**Fig. 10:**
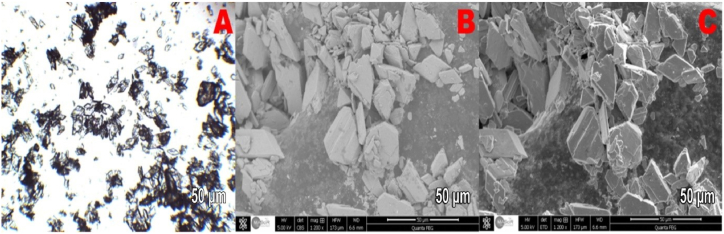
Optical microscopy and SEM images of the produced gypsum crystals without additives.

**Fig. 11:**
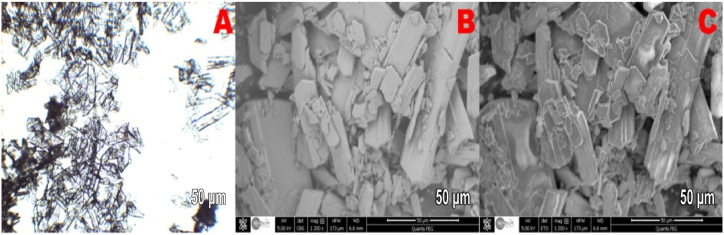
Optical microscopy and SEM images of the produced gypsum crystals with 3.6 kg/T of $P_{2}O_{5}$ of silica byproduct.

**Fig. 12:**
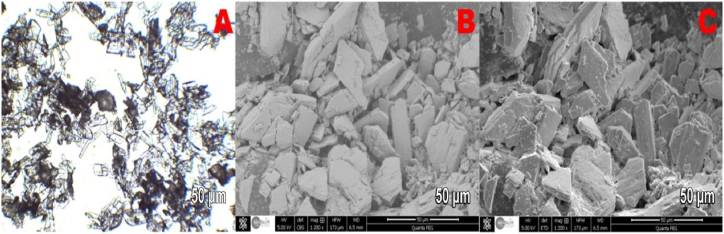
Optical microscopy and SEM images of the produced gypsum crystals with more than 3.6 kg/T of $P_{2}O_{5}$ of silica byproduct.

Fig. 10 (A – C), 11 (A – C) and 12 (A – C) show optical microscopy images (Fig. 10) and SEM images (Fig. 11, Fig. 12) of the produced PG crystals with and without the addition of the silica byproduct. These images were collected to assess the effects of the additives on the morphology of the crystals. Fig. 10A, B and 10C show that the shape of the PG crystals in the absence of additives were needle-type crystals with a high length to width aspect ratio. Such crystals are generally difficult to separate during the PG filtration process for WPA production. The SEM images of the gypsum crystals with a silica content of 3.6 kg per ton $P_{2}O_{5}$ are shown in Fig. 11. Most of these crystals have a tubular shape as it was previously described by Linnikov [48], with a length equal to double their width. It has already been demonstrated by Cocheci [49] that the presence of silica (0.93 %) leads to an increase in the length to width ratio of PG crystals. In this study, the average aspect ratio nearly tripled with the addition of the silica byproduct. This means that thicker and larger crystals were formed that could be better filtered and therefore increased the overall efficiency of the WPA production process. Further increasing the amount of byproduct silica to concentrations exceeding 3.6 kg per ton $P_{2}O_{5}$ did not further improve the shape of the PG crystals as shown in Fig. 12.

### 3.4 Economic and environmental considerations

Yu and Liu [50] convincingly point out that spent vanadium catalysts from sulfuric acid production are presently a waste product that can take up relevant amounts of land resources when stacked. A practice that can even cause environmental pollution. Sourcing vanadium from secondary sources is an active field of research and Petronikova et al. [51] provide an excellent review that indicates that recycling from spent catalysts is presently the most researched source for vanadium recycling. Despite these efforts, spent vanadium catalysts are presently still considered a waste product in Morocco, which means that they can be sourced inexpensively and if used as an alternative source of silica replace other commercial additives that are more costly. Utilizing spent vanadium catalysts would reduce costs and environmental risks associated with long-term stacking and can thus be considered advantageous. This view obviously assumes that no relevant secondary waste is generated during spent vanadium catalyst dismantling and recycling. An assumption that goes beyond the limits of this laboratory work and that will have to be reinvestigated if spent vanadium catalysts from sulfuric acid production would indeed be utilized during WPA on industrial scale.

## 4 Conclusion

In this work the impact of the addition of different concentrations of inexpensive byproduct silica from vanadium catalyst recycling during processing of Khouribga phosphate rock to WPA was investigated. The results indicate that the addition of 3.6 kg catalyst waste material that contains 47.2 % reactive silica per ton P_2_O_5_ leads to a significant increase of reaction yields and P_2_O_5_ recoveries, a better reaction efficiency (5 %), an increase in the filtration rate as well as an enhancement of 6 % of the quality of the produced WPA. The catalyst waste shows improvements similar to those of other more expensive commercial additives such as silica, perlite, clay and kaolin that are used industrially. Since the catalyst waste is significantly less expensive than the commercial silica sources currently used, we recommend further testing the process described here on larger pilot scale with the intention of implementing it in industrial WPA production.

## Data and code availability statement

Data will be made available on reasonable request. There was no specific code used for this research.

## Funding details

This research did not receive any specific grant from funding agencies in the public, commercial, or not-for-profit sectors.

## Disclosure statement

The authors report there are no competing interests to declare.

## CRediT authorship contribution statement

**Islaam Anouar:** Writing – review & editing, Writing – original draft, Methodology, Investigation, Formal analysis, Data curation, Conceptualization. **Rim Jouraiphy:** Writing – review & editing, Investigation, Formal analysis. **Hamid Mazouz:** Writing – review & editing, Project administration, Methodology, Investigation, Formal analysis, Conceptualization. **Nils Haneklaus:** Writing – review & editing. **Samia Yousfi:** Writing – review & editing, Investigation, Formal analysis. **Mouna L. Bouamrani:** Writing – review & editing, Methodology, Investigation, Formal analysis, Conceptualization.

## Declaration of competing interest

The authors declare that they have no known competing financial interests or personal relationships that could have appeared to influence the work reported in this paper.
